# Comparing the effect of auricular acupressure and body acupressure on pain and duration of the first stage of labor: study protocol for a randomized controlled trial

**DOI:** 10.1186/s13063-019-3896-0

**Published:** 2019-12-23

**Authors:** Zainab Alimoradi, Farideh Kazemi, Mahboubeh Valiani, Maryam Gorji

**Affiliations:** 1 Social Determinants of Health Research Center, Research Institute for prevention of Non-Communicable Diseases, Qazvin University of Medical Sciences, Qazvin, Iran; 2 Department of midwifery, Mother & Child Care Research Center, School of Nursing and Midwifery, Hamadan University of Medical Science, Front of Mardom Park, Shahid Fahmideh blv., Hamadan, 65178-38698 Iran; 3 Nursing and Midwifery Care Research Center, Faculty of Nursing and Midwifery, Isfahan University of Medical Sciences, Isfahan, Iran; 4 Velayat Clinical & Educational Hospital, Qazvin University of Medical Science, Qazvin, Iran

**Keywords:** Auricular acupressure, Body acupressure, Labor pain, Labor duration

## Abstract

**Background:**

Labor pain is one of the leading causes of fear of childbirth. Acupressure is a non-pharmacological pain relief method that has shown promising results in relieving this pain. The present study is designed to compare the effects of body acupressure at multiple points and auricular acupressure on the pain and duration of labor.

**Methods/design:**

In a randomized controlled trial, 90 primigravida women who attend for childbirth will be randomly assigned to one of three groups (intervention groups of either body acupressure or auricular acupressure; control, consisting of routine care). Computer-generated six-block randomization techniques will be used to determine the allocation sequence with a 1:1:1 ratio. To hide the allocation, the type of intervention will be written according to the generated sequence and put in opaque envelopes; these as well as questionnaires will be encoded. The pain score for all participants will be measured at the peak uterine contraction at 4-cm cervical dilation and at 10-cm dilation based on a visual analog scale (VAS). The duration of the active phase of labor in these groups will be recorded too. Data will be imported into SPSS-16 software. First, normality of the data distribution will be investigated. To compare labor duration among the research groups, ANOVA will be used, which will be followed, in case of significance, by the Scheffe post hoc test. Furthermore, Chi-squared test will be used to compare the categorized demographic variables and ANOVA or Kruskal–Wallis tests will be used to compare the quantitative variables in the studied groups. A significance level of 0.05 is considered significant.

**Discussion:**

In this study the effect of auricular acupressure and body acupressure on pain and duration of first stage of labor will be compared.

**Trial registration:**

Iranian Registry of Clinical Trials, IRCT20180218038789N1. Registered 2018-03-04; pre result.

## Background

Labor pain is one of the most intense pains experienced by women during their lifetime [1, 2]. Labor pain is caused by the interaction of physiological factors, such as uterine contractions and cervical dilation, and psychological factors, such as fear and anxiety [3]. Labor pain engenders an experience that most women tend to avoid and is always a source of anxiety and distress for pregnant women [4]. A considerable proportion of C-section (CS) deliveries have been performed merely due to mothers’ fear of labor pain [5].

Labor pain relief methods are divided into two major groups, namely pharmacological and non-pharmacological [6]. The most common technique for relieving pain of labor is the use of medications. However, the potential side effects of pharmacological methods for the fetus and mother have resulted in a growing interest in the use of non-pharmacological labor pain relief techniques [7].

Acupressure is a non-pharmacological pain relief method based on the principles of traditional Chinese medicine. Several acupressure points on the body are for the progression of birth and reduction of labor pain; their stimulation is believed to induce stimulation of uterine contractions and, consequently, progression of birth, on the one hand, and balance of energy as well as reduction of labor pain on the other [8]. *Sanyinjiao* (SP6), *Taichong* (LV3), *Ciliao* (BL32), *Weishu* (BL21), *Shangliao* (BL31), and *Hogu* (LI4) are among the numerous points used in acupuncture and acupressure for labor induction and management [8]. Various studies have reported reduced labor pain achieved by stimulation of a single point or a combination of two points [9–12]. GB21, BL32, LI4, and SP6 are the major points commonly proposed for enhancing uterine contractions, hard and prolonged labor, and dropping improvement. Results of an observational study of women receiving acupuncture as part of their antenatal care showed a 35% reduction in the use of labor induction, a 31% reduction in epidural analgesia, a 9% increase in the natural birth success rate, as well as shorter duration of labor compared to the local population rates [13]. Moreover, in a systematic review conducted to investigate the effect of acupressure on the onset and duration of labor, Mollart et al. reported acupressure could significantly reduce labor duration in the intervention group compared to the standard care and control groups [14]. Theoretically, when labor is slow and contractions are not intense enough, cervical dilation will be slow as well. Stimulating acupoints (acupuncture and acupressure points) can yield a balanced labor by adjusting the contractions, with improved contractions leading to reduced labor duration [15].

Auriculotherapy (AT; also known as auricular therapy) is a branch of acupressure spreading throughout the world. AT is based on a long-standing tradition and was modified and updated by Dr. Paul Nogier. Also, the World Health Organization considers AT a form of microacupuncture that can affect the whole body [16]. AT comprises several types of therapy, including auricular acupuncture, electro-stimulation, and acupressure. Various studies have reported promising results when using AT for the control of pain caused by backache, [17] pelvic fractures, [18] dysmenorrhea, [19–21] and polycystic ovarian syndrome [22]. However, only a limited number of studies have been done on the effect of auriculotherapy on labor pain, and these have yielded inconsistent results. Accordingly, in Rastegarzadeh et al.’s work, auriculotherapy led to a significant reduction in labor pain among nulliparous women [23], whereas a study by Maftoni and Shimo indicated that use of auriculotherapy caused no significant reduction in the studied women’s labor pain [24].

As can be inferred from investigations, most of the studies on body acupressure have more frequently applied pressure on one or two points; only a few studies have focused on a combination of points affecting the pain and progress of labor. Furthermore, some studies have been conducted on the effect of auricular acupressure on the reduction of different pain throughout the body, the results of which have confirmed the effectiveness of this technique. However, only a few studies have addressed the effect of auricular acupressure on labor pain, yielding inconsistent results [3, 4]. Given the small number of studies on the effect of body acupressure at multiple points and auricular acupressure on the pain and duration of labor, we designed the present study. In this clinical trial, the primary objective is to compare the effects of auricular acupressure, body acupressure, and routine treatment on the pain score at the time of 4-cm and 10-cm cervical dilations. The secondary objective is to investigate the effects of the described interventions on the duration of the first stage of labor, which is defined as the time interval between the 4 cm and 10 cm cervical dilations. This is a single-center, parallel, double blinded randomized controlled trial to investigate the superiority of auricular acupressure and body acupressure compared with routine care during the first stage of labor. The allocation ratio for the groups is 1:1:1.

## Methods/design

### Research setting and design

The present randomized controlled trial with parallel control group will be conducted on 90 pregnant women referring to Kowsar Hospital in Qazvin for labor that agree to participate in the study and meet the inclusion criteria. Qazvin is the largest city and capital of the province of Qazvin in Iran, located 150 km (93 miles) northwest of Tehran at an altitude of about 1800 m (5900 feet) above sea level. This protocol is organized based on SPIRIT guideline. This writing guideline provides Standard Protocol Items: Recommendations for Interventional Trials. The SPIRIT checklist for present study is provided in Additional file 1. 

### Intervention and comparator

**Fig. 1:**
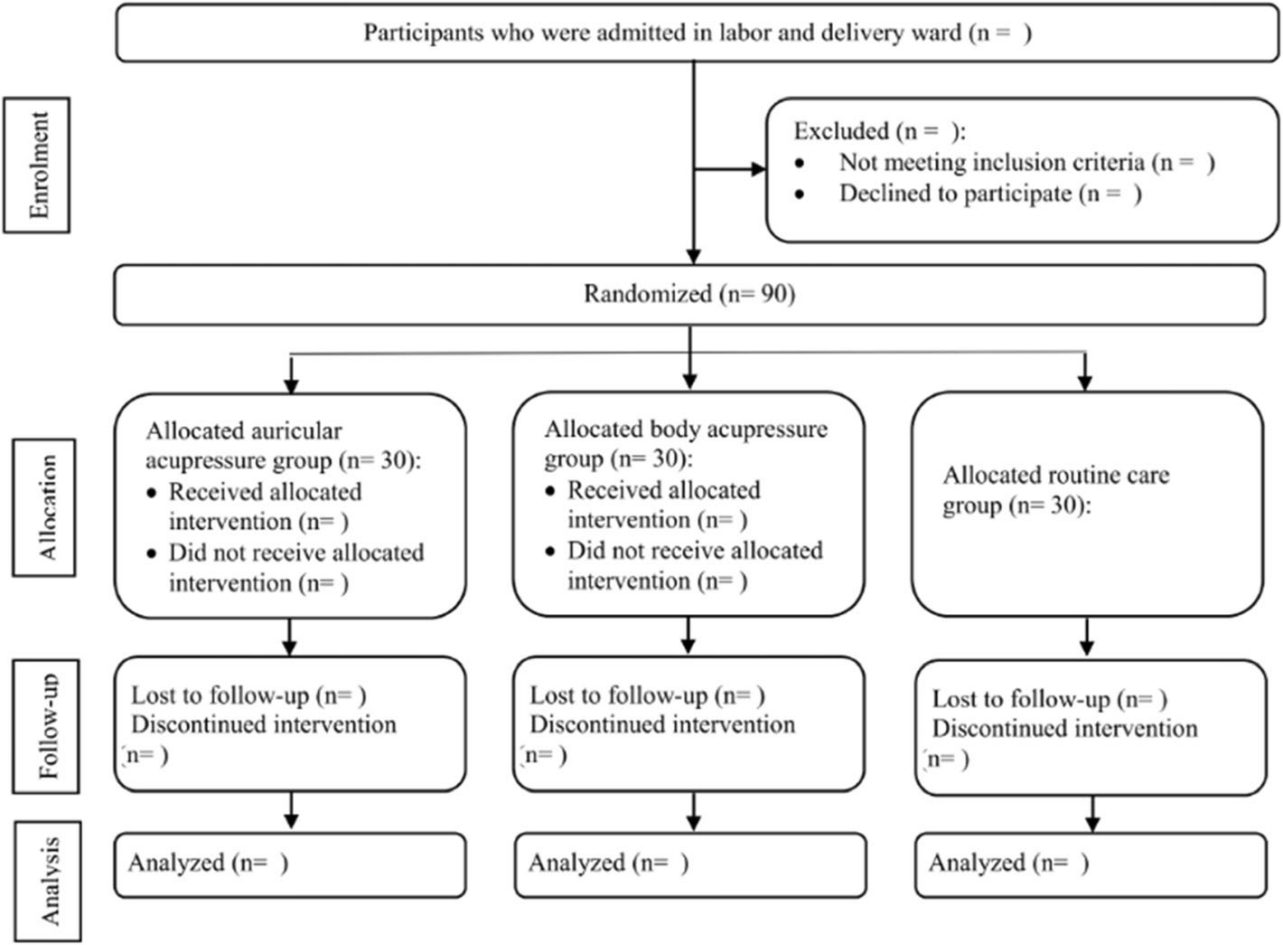
Trial flow overview

The participants are divided into three groups, body acupressure (intervention 1), auricular acupressure (intervention 2), and routine care (comparison) groups (Fig. 1).

### Inclusion and exclusion criteria

#### Inclusion criteria

The inclusion criteria in the present study are primigravida women aged 19–35 years at gestational age of 37–42 weeks with a singleton pregnancy, cephalic presentation, no history of chronic diseases such as diabetes, cardiovascular disease, hypertension, hepatic and renal disorders, etc., a lack of pregnancy complications such as preeclampsia, gestational diabetes, bleeding, etc., a height above 150 cm, and admission at the beginning of the active phase (3–4 cm dilation).

#### Exclusion criteria

Refusal to continue participation in the study, receiving pain relief drugs 3 h before or during the study, labor induction or enhancement by medication, and emergency CS birth constitute the exclusion criteria of this study.

### Informed consent process

The first author will explain the research objectives and method to pregnant women meeting the inclusion criteria at the time of their admission to the labor and birth ward. The first author will then obtain written consent from patients willing to participate in the trial.

### Sample size estimation

To calculate the minimal clinically important difference (MCID) for the duration of the first stage of labor and the pain score, we used previous study data which examined the effect of auricular [24] and body [25] acupressure on labor pain intensity and labor duration. Using the method of Wyrwich et al. [26], MCID score for significant pain reduction during labor was one score based on visual analogue scale. Using the G*Power software 3.1.9.2, α = 0.05, and power = 0.80, considering SD = 1.4 and M0 = 7.2 for the labor pain score and 10% drop out of subjects, the sample size was calculated to be 30 patients for each group.

### Randomization and blinding

A person not involved in the study will use the computer-generated six-block randomization technique to determine the allocation sequence with a 1:1:1 ratio. In order to ensure allocation concealment, this person will write the type of intervention based on the predetermined sequence and put it in an opaque envelope; these will then be encoded. The questionnaires are encoded as well. Accordingly, for a participant receiving the intervention in a pocket encoded with “1”, a questionnaire with the same code will be completed. To observe blinding during data collection, one of the coworkers will perform the intervention plan and another coworker who knows nothing about the intervention will collect the data. Finally, once the questionnaires are gathered and their data are imported into SPSS-16 software, it will be determined which codes should be assigned to group A and which codes to group B or C. Afterwards, analysis of the data will be performed. The person performing the data analysis will also be blind to the type of intervention used for each group (Fig. 1).

### Follow-up

#### Assessments

The duration of the first stage of labor is determined in minutes and written in the questionnaire. To assess the pain score, pain measurement using a VAS (visual analogue scale) ruler will be used at 4- and 10-cm dilations. Furthermore, the midwifery and demographic characteristics questionnaires are completed after finishing the birth.

#### Administration of intervention

Selection of acupoints is based on ear and body microsystems and meridians. Some treatment plans include a combination of some acupoints for every problem. These selections of points were originally derived from treatment plans developed in China, but modified by auriculotherapy discoveries in Europe and the United States. Theoretically, every acupoint can exert some identified effect for the selected condition [27–30]. For the present research, a set of primary and master acupoints was selected. Using combination sets of points is proposed to be more effective than single points. As there is scant evidence on the efficacy of applying multiple acupoints, we aim to investigate the efficacy of multiple acupoints on labor pain and duration.

**Fig. 2:**
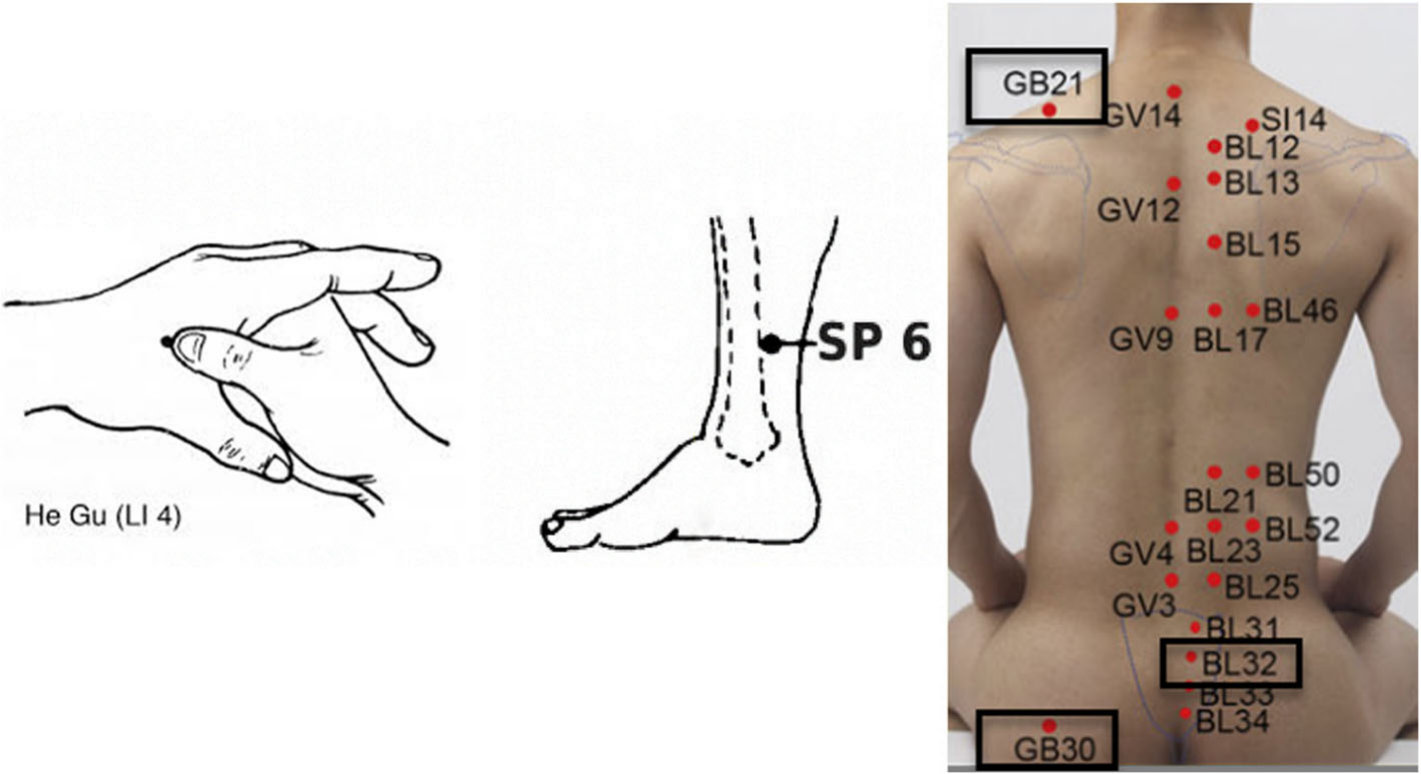
Body acupoints

In the body acupressure group, pressure is applied to the GB30, GB21, BL32, LI4, and SP6 points [13, 29] (Fig. 2) by a researcher who has been well trained for this purpose. The GB21 point has an action of release and descent, which is purported to facilitate fetal descent in the active phase and second stage of labor [31]. Stimulating the LI4 point is effective for reduction of labor pain and promoting stronger and/or more coordinated contractions [9, 32–34]. Stimulating BL32 is effective for the reduction of labor pain and induction of labor [35, 36]. One of the most important and frequently used points for obstetric and gynecologic concerns is SP6. Indications of this point include labor augmentation, for irregular contractions to encourage efficient labor and reducing a persistent cervical lip [37–39], reduction of labor pain [34, 38–41], and reduction of the length of labor [34, 38]. To perform the acupressure on the specified points on the body, each point will be pressed by the thumb for 2 min, so that one-third of the nail bed becomes white. Pressure to these points would be applied at 4, 6, and 8-cm dilations.

**Fig. 3:**
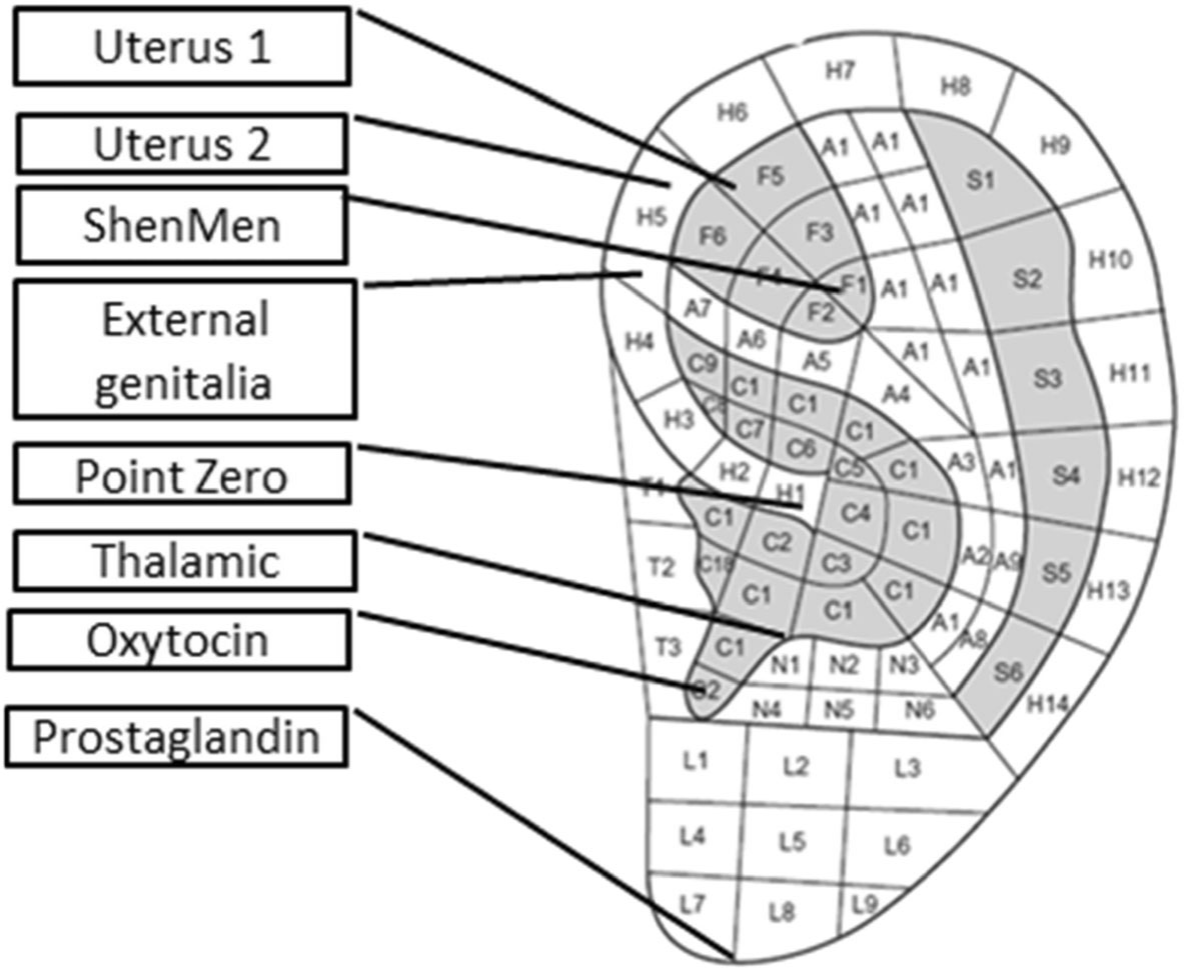
Ear acupoints

**Fig. 4:**
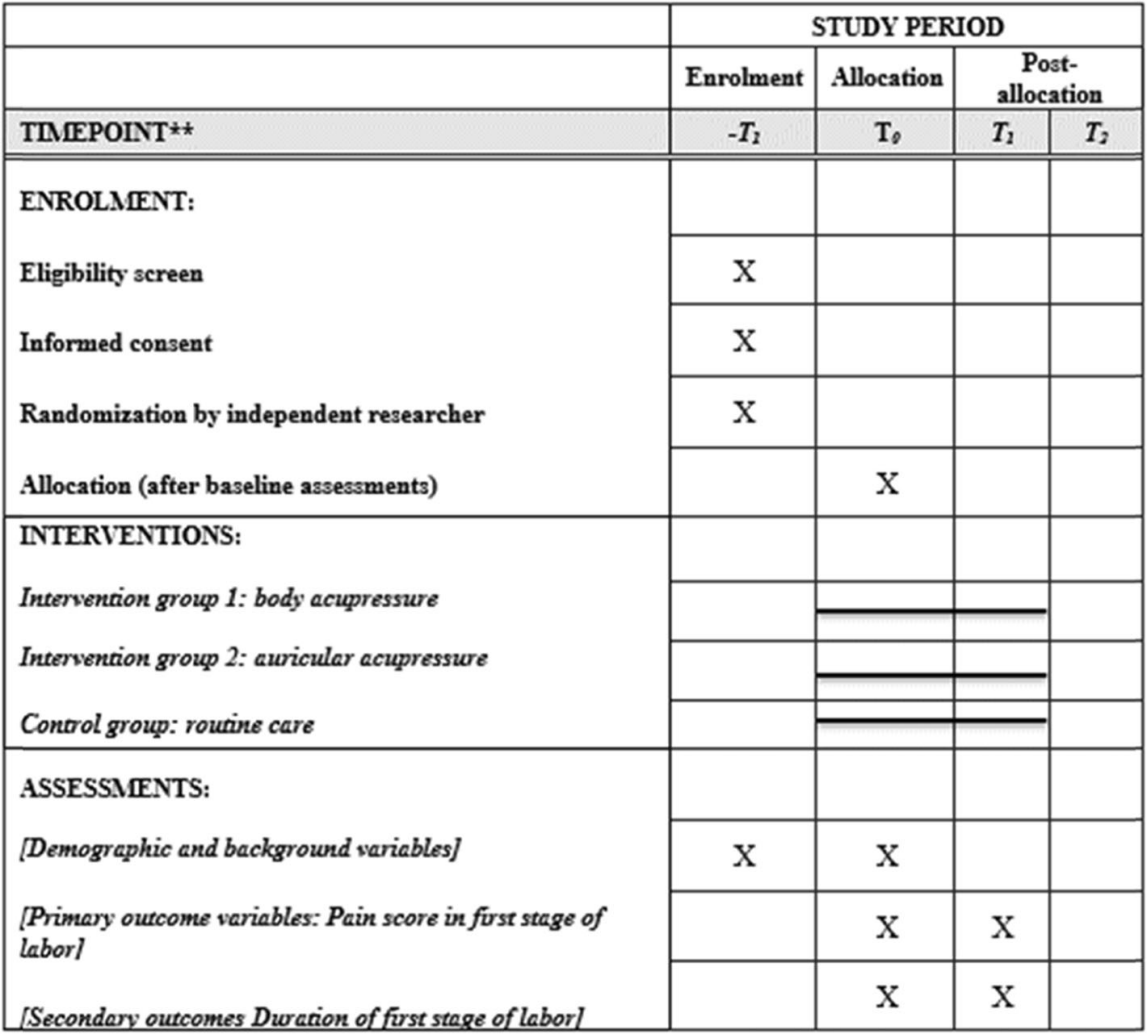
SPIRIT Schedule of enrolment, interventions, and assessments

In the auricular acupressure group, pressure will be applied to the master auricular points on the external ear (point zero, Shen Men point, and thalamic point) and primary auricular points (uterus points 1 and 2, external genitalia point, oxytocin and/or prostaglandin points) [27] by a well-trained researcher. Ear acupoints are shown in Fig. 3. The master points are so identified because they are typically active in most patients and they are useful for the treatment of a variety of health disorders. Point zero brings the whole body toward homeostasis, producing a balance of energy, a balance of hormones, and a balance of brain activity and is frequently combined with the Shen Men point for treatment of most health disorders. The purpose of Shen Men is to tranquilize the mind and to facilitate a state of harmony, serenity, and a deeper connection to one’s essential spirit. This master point alleviates stress, pain, tension, anxiety, depression, insomnia, restlessness, and excessive sensitivity. The thalamic point affects the relay of sensory information to the cerebral cortex and modulates hypothalamic regulation of autonomic nerves and endocrine glands. It is also used for alleviating most pain disorders, both acute and chronic. Primary auricular points are the most effective set of auricular points for the treatment of a health disorder in a particular body organ or for a physiological dysfunction [27]. The given pressure will be applied using an adhesive containing auriculotherapy-specific Vakharia seeds. Furthermore, auricular stimulation at the specified points will be performed every 30 min. It should be noted that the comparator group will receive routine care (Fig. 4).

### Intervention fidelity

One of the researchers (MG) responsible for intervention is trained to perform auriculotherapy. Training and monitoring of her performance on implementing the intervention will be carried out by MV, who is an expert in this field.

### Primary outcome measurements

#### Pain score in the first stage of labor

In the present study, the pain score (pain intensity) at 4- and 10-cm dilations is measured in all three groups using a VAS and then compared.

### Additional outcomes

#### Duration of the first stage of labor

The present study is aimed to determine whether auricular acupressure and body acupressure can reduce the duration of the first stage of labor in nulliparous women compared to routine care.

#### Data collection and storage‌

Based on the predetermined allocation sequence, the studied women are divided into three groups, auricular acupressure, body acupressure, and routine care groups. The pain score for all participants will be measured at the peak uterine contraction at 4-cm cervical dilation and then recorded in the questionnaire. Subsequently, at 10-cm dilation, the pain score of all participants in the three groups will be re-measured and recorded. Furthermore, in order to investigate the duration of the active phase of labor in these groups, the onset and termination times of the active phase of the first stage of labor will be recorded in the questionnaire. The difference of these two recorded times indicates the duration of this stage. The remaining parts of the questionnaire are completed after the birth. Ethics committee will monitor the whole procedure including data gathering.

### Data analysis

Once collected, the data are imported into SPSS-16 software. Firstly, normality of the data distribution will be investigated (via three methods, including Kolmogorov–Smirnov, histogram, and dispersion and central indices) and if the distribution of variables is not normal, then we will use an appropriate transformation. To compare the labor duration and pain score among the research groups, one way ANOVA test will be used, which will be followed, in case of significance, by Scheffe post hoc test. If there are potential confounding variables, a multiple linear regression test will be used. We will check for the assumptions and concerns of the regression model. Furthermore, Chi-squared test will be used to compare the categorized demographic variables in the studied groups. In order to compare the quantitative variables, in case of normal and abnormal distribution of the variables, ANOVA and Kruskal–Wallis tests will be used, respectively. The post hoc of these tests will be reported if needed. The study will be analyzed using an intention-to-treat (ITT) approach and using a multiple imputation strategy to account for missing outcomes in ITT. Also, apropos of the significance level, 0.05 is considered significant.

### Reporting of adverse events

Any kind of unwanted outcome in the participants will be reported.

### Patient and public involvement

Patients and the public were not directly involved in the development of this study protocol. However, the development of the research question and outcome measures is in accordance with previous published studies on patients’ priorities, experiences, and preferences. We will disseminate results to the study participants through journal publication as well as research conferences.

## Discussion

Improving the maternal and neonatal health is one of the sustainable development goals world-wide which Iran's Minestry of Health has strategic planning to reach this goal, too. Achieving these goals involves reducing the maternal and neonatal mortality rate due to complications of pregnancy and delivery, reducing the use of CS without indication and promoting normal delivery [42]. One way to promote natural childbirth is a painless labor. The American College of Obstetricians and Gynecologists has confirmed that the request for pain relief from the patient is indicative of the need for pain relief [43]. Labor pain relief methods are divided into non-pharmacological methods and pharmacological methods. Hypnosis, acupuncture, therapeutic touch, relaxation, massage therapy, and music therapy are non-pain relief methods [6]. Systemic medications, inhalation anesthesia, local anesthesia and general anesthesia are pharmacological methods of pain relief [43].

Based on available scientific resources, massage can reduce labor pain by the gate control mechanism of pain. In this way, massage activates large neural fibers and closes the gates of pain transfer. Another theory in this area is that massage may release endorphins and thereby reduce the pain [44]. In this study we will compare the effect of auricular acupressure and body acupressure on labor pain. In addition, we will study the effect of the two noted methods on duration of first stage labor.

### Trial status

Recruitment began in August 2018 and is ongoing. Data collection will probably be completed in December 2019.

## Supplementary information

**Additional file:** **Appendix 1:** SPIRIT checklist..

## Data Availability

Original research data can be requested from the corresponding author. Additional files: Appendix 1: SPIRIT checklist.
